# Acceptability and Feasibility of Provision of COVID-19 Services by Community Health Workers to Remote Gold Mining Communities in Suriname

**DOI:** 10.4269/ajtmh.23-0734

**Published:** 2024-08-27

**Authors:** Stephen Vreden, Marieke Heemskerk, Hélène Hiwat, Hedley Cairo

**Affiliations:** ^1^ Foundation for the Advancement of Scientific Research in Suriname, Paramaribo, Suriname;; ^2^ Social Solutions, Paramaribo, Suriname;; ^3^ Malaria Program, Ministry of Health Suriname, Paramaribo, Suriname

## Abstract

Gold mining communities in the Amazon region typically have limited access to public health services. In Suriname, the Ministry of Health Malaria Program (MoH-MP) works with community health workers (CHWs), people from mining communities without a formal medical degree, to provide malaria diagnostic and treatment services. During the COVID-19 pandemic, the MoH-MP trained 21 of these CHWs in COVID-19 outreach and testing, using rapid antigen tests for symptomatic persons in their communities; afterward, a mixed methods research approach was used to investigate whether including COVID-19 services in the tasks of the CHWs was feasible and accepted among gold mining populations. Also, CHWs took part in active case detection missions to proactively offer COVID-19 testing to all inhabitants of specific mining areas, regardless of symptoms. In the 6 months of field implementation (May–October 2022), 1,300 persons were tested for COVID-19, among whom 28.7% were women. Eight percent tested positive. Of the 312 asymptomatic persons tested, 2.2% tested positive. Qualitative semi-structured interviews with the CHWs and quantitative pre- and postintervention surveys revealed that the communities appreciated the nearby and free COVID-19 testing opportunity. The intervention motivated individuals who otherwise would not have been tested to test for COVID-19. Twenty-nine percent of those who had tested at least once for COVID-19 reported that their most recent test was conducted through the services of the CHWs. The results suggest that integrating COVID-19 testing into other CHW services can lower health access barriers in difficult-to-reach populations in remote communities.

## INTRODUCTION

In many countries, particularly in the global south, community health workers (CHWs) dramatically improve health outcomes, reduce healthcare costs, and bridge the gap in health communication between healthcare providers and their clients.^1^ These clients often include difficult-to-reach populations who have less than optimal access to regular public health services for a variety of reasons, including the health clients’ remoteness, language barriers, lack of access to transportation, and distrust of regular healthcare providers.^2^^,^^3^ This case study analyzed the efficiency and added value of using CHWs to provide coronavirus disease 2019 (COVID-19) services to mobile migrant mining populations in the Amazon region.

**Figure 1.**
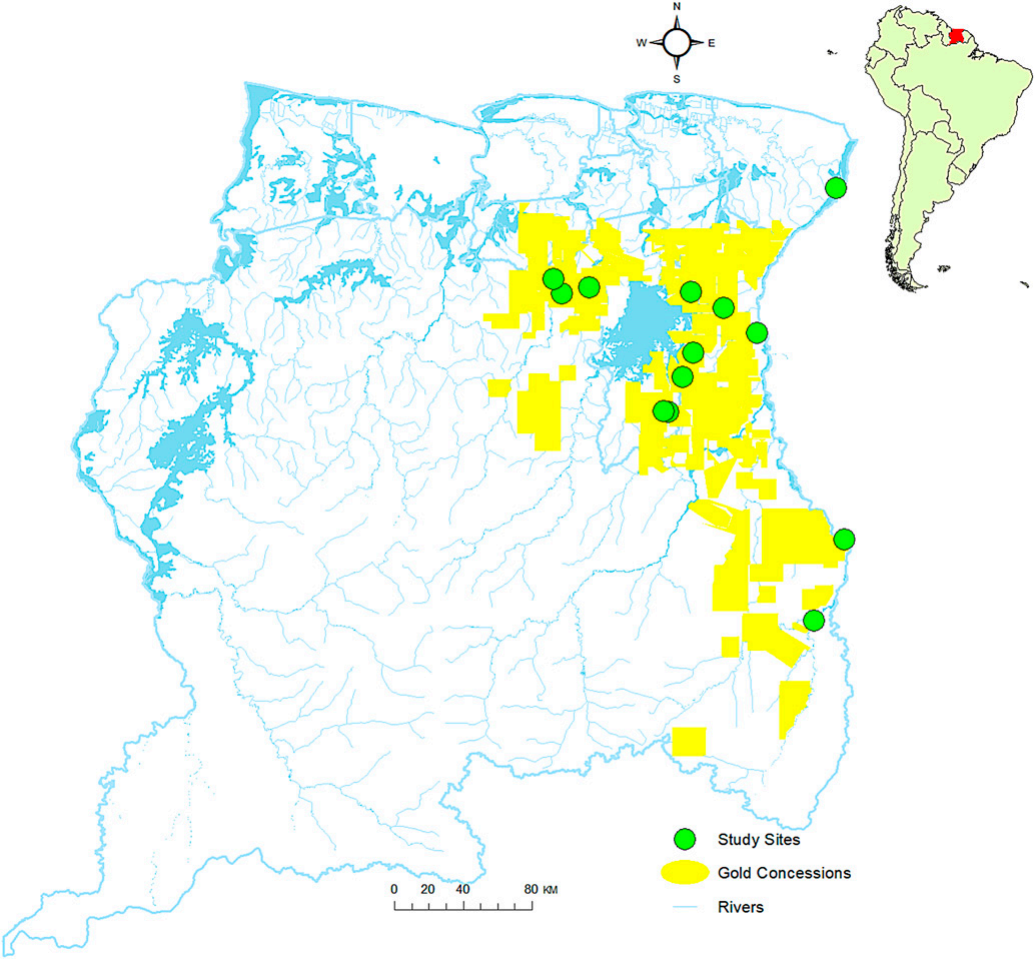
Map of Suriname on which locations of small-scale artisanal gold mining sites in the interior of Suriname are indicated, including sites where COVID-19 testing was carried out.

This paper focuses on the small Amazon country of Suriname situated on the northern coast of South America, bordered by French Guiana to the east, Guyana to the west, and Brazil to the south (Figure 1). Eighty-six percent of the estimated population of 616,500 lives in urban coastal areas.^4^ The remaining 14.2% lives in Maroon and Indigenous communities in the interior of Suriname.^4^ In addition, an estimated 20,000 small-scale gold miners work and live in Suriname’s interior. About two-thirds of the small-scale gold mining population consists of Brazilian migrants. Others are local Surinamese and smaller numbers of migrants from other countries such as China, the Dominican Republic, Cuba, and Guyana.^5^

**Figure 2.**
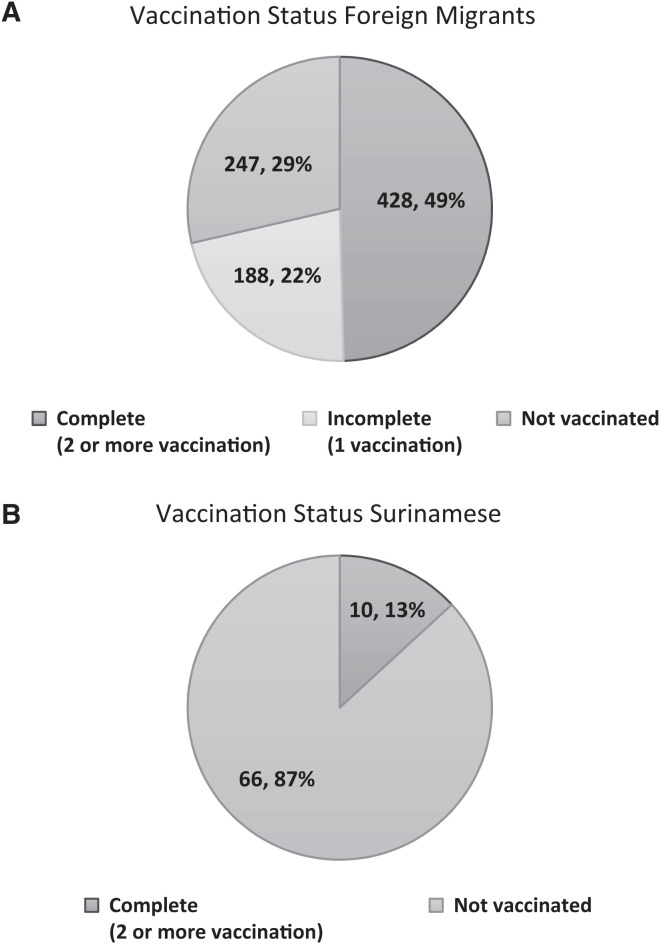
Vaccination status of the foreign (**A**) and Surinamese (**B**) participants from the gold mining communities who underwent COVID-19 antigen testing by community health workers during the intervention.

Health inequities in Suriname exist in relation to geographic location, socioeconomic status, population group, and sex.^6^ A specifically underserved group consists of small-scale gold miners working in faraway areas in the forested interior of the country.^6^ These small-scale gold mining populations are highly mobile; they often move from place to place when gold earnings in one place diminish and stories of new gold prospects elsewhere circulate.^7^ In the gold mining areas, they live in more or less stable camps in the forest or in gold miners’ settlements. In these miners’ settlements, there is no government or private clinics.^6^ The high expenses related to traveling to health service providers and the (resulting) high propensity to self-medicate further motivate the limited use of formal public health services by small-scale gold mining populations in these regions.^6^^,^^8^

In the 1990s and 2000s, the limited availability of malaria services (outreach, diagnosis, and treatment) in remote gold mining areas and the pivotal role of these areas in spreading malaria to traditional communities were of particular concern. The Ministry of Health Malaria Program (MoH-MP), with the support of national and international partners, was able to implement a new strategy that focused on reaching key risk populations and implementing innovative approaches for hard-to-reach populations. Within this context, a network of CHWs for malaria was established.^9^^,^^10^ This people-centered approach proved very effective and has been recognized within the region as a “best practice” for service provision among mobile and hard-to-reach populations.

In the initial phase of the COVID-19 pandemic, reliable diagnosis of COVID-19 could only be done by use of polymerase chain reaction. For people living in remote areas, this required collection of nasopharyngeal swab samples that had to be transported to an urban laboratory; therefore, results only became available after at least 2 days. Also, because of the remoteness of the mining areas, daily swabbing services were not feasible. When reliable point-of-care rapid diagnostic tests (RDTs) became available, testing for COVID-19 became much easier. However, because of the absence of laboratory staff in the mining fields, the target population could still not benefit from this improvement. To reduce inequities in access to COVID-19 services, the MoH-MP implemented a public health intervention that included an anthropological study to assess whether the inclusion of COVID-19–related health services in the tasks of existing CHWs who delivered malaria services in the gold mining areas was feasible and would be accepted by both the target population and the CHWs.

The public health intervention and study had the following three objectives:

1. To assess whether continuous availability of COVID-19 diagnosis in remote, mobile, migrant gold mining communities by local CHWs engaged by the MoH-MP and trained in using severe acute respiratory syndrome coronavirus 2 (SARS-CoV-2) antigen RDTs (Ag-RDTs), was feasible
2. To assess how this intervention was received by both the CHWs and the target population
3. To measure intervention impacts on the perceptions and behavior of targeted gold mining populations

## MATERIALS AND METHODS

The research study consisted of two components: a public health intervention and an anthropological study to measure the impact of the intervention. For the anthropological study, data were collected in May 2022 (preintervention) and November 2022 (postintervention). In each period, anthropological data collection consisted of a quantitative survey with inhabitants of gold mining communities and qualitative, semi-structured interviews with the CHWs who participated in the COVID-19 program. The Suriname Ministry of Health’s Ethics Advisory Board (CMWO) provided ethical approval for the research study (MOH040222, approval obtained February 4, 2022).

### Public health intervention.

In March/April of 2022, after a general assessment of their personal attitude toward COVID-19 and their preparedness to participate in the study, 21 CHWs were trained by the MoH-MP in the delivery of COVID-19 services to remote gold mining communities in the Suriname interior. The MoH-MP recruited these CHWs from the group that was already living in gold mining communities and providing malaria services in the mining areas. Additional inclusion criteria were that, based on the general assessment, the CHWs had shown intrinsic motivation to perform COVID-19 services for their communities and were willing to conduct the COVID-19 rapid tests (nasal swabs). The training curriculum included basic knowledge of COVID-19 transmission and prevention; symptoms; clinical evaluation (a short questionnaire, assessment of body temperature, breathing frequency, oxygen saturation, danger signs); safely taking nasal swabs; carrying out antigen tests; instructions on isolation; quarantine and contact tracing; reporting to the supervisor; and inquiring about vaccination preparedness. The training was carried out by medical professionals of the Academic Hospital Paramaribo, supported by laboratory staff who were proficient in COVID-19 diagnostic training. Field-workers who conducted, the social study were trained by the social scientist involved in the study (M. Heemskerk).

Training materials were developed in working group sessions by the authors. Community health workers were instructed to offer testing to all persons in their area of service who had symptoms that could be attributed to COVID-19, including those in whom a malaria infection was suspected, and also to the recent contacts of persons who tested positive. Ultimately, for several reasons (resignation, pregnancy, traveling abroad, etc.) only 16 CHWs were involved in the study. During active case detection (ACD) campaigns for malaria, people who were tested for malaria were offered a COVID-19 antigen test if they had symptoms. Furthermore, the CHWs were trained to provide general COVID-19 community education and prevention activities. Community education was carried out in focus group discussions, in which people’s thoughts and practices were discussed and corrected in an explanatory way, and reasons and methods of infection prevention were explained. Perceived risks that arose from charging laypeople with the task of taking nasopharyngeal swabs were mitigated by allowing CHWs to take part in the study only after ensuring that the procedure was carried out according to professional medical instructions. To verify the quality of their performance, all CHWs were observed at least once per month while carrying out their tasks and, if necessary, received instructions.

There are hundreds of small-scale artisanal gold mining sites located in the forest of Suriname. In this study, which was intended to be a pilot study, we randomly selected four sites with at least a hundred people that were remote from health facilities in the country or cross-border. Health data were recorded on paper forms and then entered into Excel files. People who produced health data and those who had access to the data were asked to declare that no information would be shared with anyone not involved in the research study. The data were stored on a local (isolated) personal computer. For data analysis, no specific statistical analysis program was used; rates, age ranges, etc., were extracted from the Excel file.

### Diagnostic data.

All persons for whom a malaria test was clinically indicated were at the same time offered SARS-CoV-2 Ag-RDT. In addition, people with other symptoms that could be attributable to COVID-19 were also offered a COVID-19 antigen test. The CHWs recorded every case that was offered an antigen test, as was already the custom for malaria. Whenever the community reported that there were people with flulike symptoms in one of the dwellings in the catchment area of CHWs, they traveled to that dwelling for extensive testing of the people there. Positive results were reported on a daily basis to the supervisor by phone or, if the internet was available, by a WhatsApp message. This allowed for continuous central monitoring of the epidemiological situation. After analyzing all reported cases, the supervisor presented a weekly overview to the Bureau of Public Health. In case of a local epidemic, reporting was immediate. The supervisor also entered all data in a separate, anonymized database.

### Anthropological study part I: Survey with inhabitants of gold mining areas.

#### Quantitative pre- and postintervention surveys and survey team.

In May 2022, prior to the public health intervention, a survey team led by an anthropologist conducted a quantitative health perception and behavior survey among gold miners and others in the mining communities, such as cooks, shop owners, and other mining survey providers. In November 2022, after the public health intervention, a similar quantitative survey took place with inhabitants of the same gold mining areas. These quantitative surveys took about 20–30 minutes and were performed in the language of choice of the respondent. This was typically Portuguese or Sranan Tongo (Suriname creole), but also occasionally Spanish or English.

During both survey periods, the survey team consisted of the same three interviewers, an experienced survey supervisor, and an anthropologist as team leader. All team members had previous experience with similar work in Suriname gold mining areas and were fluent in all relevant languages: Brazilian Portuguese, Spanish, Dutch, Sranan Tongo, and English. The surveyors received a 3-day training that covered the study aims, survey questions, consent procedure, and ethics. The team leader and/or the survey supervisor accompanied the survey team at all times to perform quality control. In the field, immediately after completing a survey, the supervisor or team leader would check the survey forms for errors, omissions, inconsistencies, or additional explanations. In such cases, the interviewer was asked to return to the interview participant to complete or correct the survey.

#### Study areas for the anthropological surveys.

For the anthropological survey, we selected gold mining areas where CHWs who had been part of the COVID-19 diagnosis and services training were working. Two main gold mining regions were selected: the area south of the hydropower lake with the main mining communities Agua Branca and Curutela de Claudia and the area west of the lake with the most important gold mining village, Vila Brazil (Figure 1). Within these two larger areas, we targeted three different mine sites. Given our earlier work in these areas, we are confident that the selected sites captured the diversity of experiences in Suriname gold mining areas.

Each selected gold mining area consisted of one or more *curutelas*, or mining communities, and scattered mining camps. These mining camps in the forest typically house between 10 and 20 persons who work together in one operation.

#### Sampling design and inclusion criteria.

Random sampling of the mining population was difficult. Gold miners are not registered, many foreign miners are illegally in the country, and populations change in response to gold discoveries or other economic or political trends. Moreover, because of the isolation of many gold mining communities and high travel expenses, it was not possible to visit all areas.

A stratified purposive sample was taken to ensure that the experiences of women and men and of both migrant miners and local Surinamese gold miners were captured. Survey participants had to be at least 18 years of age, had to be working and/or living in a gold mining area since at least January 2021, and had to consent to participation in the survey.

A total of 235 persons participated in the preintervention survey. During the quantitative survey after the health intervention, in the exact same areas, 270 persons participated. The small difference in response rate can be explained by the presence of people willing to be interviewed during the particular period when the surveyors were in the area. Because gold miners move frequently, both between mining camps within an area and between mining areas, it was not possible to approach the exact same persons for the pre- and postintervention surveys. Reflecting Suriname’s gold mining population, the majority of persons in our sample were Brazilians (Table 1). During the preintervention quantitative survey, the median age of participating men was 46.5 years, and the median age of women was 37 years. During the postintervention survey, the median ages of men and women were 39.5 and 40 years, respectively (Table 1).

**Table 1:** Demographic characteristics of inhabitants of gold mining communities who participated in the pre- and postintervention quantitative surveys

Demographics	Preintervention	Postintervention
Sample Size, *N*	235	270
Sex, % (*n*/*N*)		
Female	40.0 (94/235)	34.4 (93/270)
Male	60.0 (141/235)	65.6 (177/270)
Nationality, % (*n*/*N*)		
Surinamese	20.0 (47/235)	15.9 (43/235)
Brazilian	72.8 (171/235)	71.9 (194/270)
Dominican	3.8 (9/235)	4.1 (11/270)
Cuban	1.7 (4/235)	2.2 (6/270)
Guyanese	1.3 (3/235)	–
Venezuelan	–	1.5 (4/270)
Chinese	0.4 (1/235)	–
Vietnamese	–	0.4 (1/270)
Haitian	–	0.4 (1/270)
Age		
Mean Age (range) Female	37.7 Years (19–60)	39.2 Years (19–65)
Median Age Female	37 Years	40 Years
Mean Age (range) Male	45.2 Years (18–67)	40.6 Years (19–73)
Median Age Male	46.5 Years	39.5 Years
Profession		
Main Profession Female	Cook (48.9% of Females)	Cook (46.2%)
Main Profession Male	Gold Miner, Laborer (61.0% of Men)	Gold Miner, Laborer (55.4%)

#### Approach of participants, consent procedure, and participant protection.

Each inhabitant of the selected gold mining areas who was 18 years or older was a potential candidate for the survey. During the survey work, the survey supervisor and/or the team leader kept count of the number of participants in subcategories to ensure that target shares of men and women and Surinamese versus migrant gold miners were reached. Each potential survey participant was approached in a nonobtrusive manner. The interviewer introduced him- or herself and explained the survey’s purpose. Next, the interviewer conducted the consent procedure, following a script that had been practiced during the interviewer training. Participants were informed that participation in the survey was anonymous and voluntary and that they had the right to refuse to participate or to withdraw at any moment during the survey. Consent was conducted orally, to account for the fact that a significant share of the target population was (semi-)illiterate.

To protect participant identity, no names or other identifying information was recorded. Nor did surveyors ask to see personal documentation, ask questions about sensitive personal issues such as residency or migration status, or take pictures without consent. The questionnaire we used for the interviews is available in Supplemental file 1.

The surveyor training included an extensive module about ethical behavior, including protection of participant identity.

#### Survey structure and content.

The survey contained mostly close-ended questions and focused on perceptions and knowledge of COVID-19 (cause, protective measures, access to care) and COVID-19 related to behavior, including testing behavior. Prior to the first field data collection trip, a pilot survey was conducted with five persons living in gold mining areas, who were in Paramaribo at the time. Based on the pilot survey results, questions were revised, added, and deleted. The pilot surveys were not included in the dataset.

Survey questions also asked inhabitants of gold mining communities about their knowledge of and experience with the CHWs. For the postintervention survey, additional questions were added about the intervention. For example, survey participants were asked whether they had received COVID-19 information from the CHWs and whether they had performed a COVID-19 test with the CHW.

#### Data entry, storage, and analysis.

Because of limited access to electricity and the internet, survey data were recorded on paper forms rather than tablets or other electronic devices. All forms were checked the same day of data collection and subsequently collected in a waterproof bag that was stored in the room of the team leader.

Survey data were entered into IBM SPSS statistics v. 22 software (Chicago, IL). SPSS software was also used for data analysis. Entered data were stored on the password-protected laptop of the team leader.

### Anthropological study part II: Semi-structured interviews with CHWs.

#### Qualitative pre- and postintervention semi-structured interviews.

Sixteen CHWs both followed the COVID-19 training and participated in the public health intervention in the gold mining communities (Table 2). Both before and after the health intervention, the anthropologist (team leader, anthropological study) and survey supervisor reached out to these CHWs to conduct qualitative, semi-structured interviews. The CHWs lived and worked in remote areas dispersed across the interior, and it was not possible to visit or speak to all of them. Some CHWs were interviewed in their working area, others when they happened to be in Paramaribo, and yet others were interviewed by phone, yet not all areas where CHWs worked had phone reach. Before the health intervention, 13 CHWs were interviewed. After the intervention, 12 CHWs were interviewed: two men and 10 women. Others could not be reached during the research periods.

**Table 2:** Data on the training of CHWs and the tests carried out in the target population

CHWs, Participants, Tests and Results	Female (%)	Male (%)	Total
Number of CHWs Trained	18 (85.7)	3 (14.3)	21
Number of CHWs Deployed	14 (87.5)	2 (12.5)	16
Number of COVID-19 Ag-RDTs Carried Out	373 (28.7)	927 (71.3)	1,300
Number of Positive Test Results	23 (22.5)	79 (77.5)	102
Number of Contacts Tested	38 (24.7)	116 (75.3)	154
Number of Positive Results in Contacts	2 (7.4)	25 (92.6)	27
Number of Asymptomatics Tested	89 (24.7)	223 (75.3)	312
Number of Positive Asymptomatics	1 (14.3)	6 (85.7)	7
Number of Referrals for Clinical Care	1	–	1

Ag-RDTs = antigen rapid diagnostic tests; CHW = community health worker.

The semi-structured interviews with CHWs typically took about 1 hour. Because most of these CHWs were Brazilian, most interviews were conducted in Portuguese. Other interviews were conducted in Sranan Tongo (Suriname creole language) or Dutch. Before the health intervention, we asked CHWs about, among others, their feeling about testing patients for COVID-19 and related concerns about becoming infected, their confidence in being able to provide adequate COVID-19 information, their motivation, and their expectations with regard to reactions from their communities. After the intervention, the CHWs were interviewed about support received from the MoH during their COVID-19 work, reactions—positive and negative—from community members, their efforts in convincing community members to test for COVID-19, and lessons learned. Interview responses were recorded in writing/typing and transferred to an Excel sheet, grouping the answers from different respondents on each question together. No coding program was used for analysis. The data were stored on a password-protected computer.

## RESULTS

### Results from semi-structured interviews with the CHWs (anthropological study, part II).

The 13 CHWs who were interviewed before the intervention were two men and 11 women. Nine persons were Brazilian, and four were Surinamese. They ranged in age from 26 to 53 years. Among the 12 CHWs interviewed after the intervention were two men and 11 women, among whom nine were Brazilians and three Surinamese.

The interviewed CHWs had very different jobs and functions in the mining communities where they worked. Both groups included individuals whose main jobs were gold miner, carpenter, equipment owner, housewife (i.e., not earning own income), public worker, pizza seller, mechanic, bar owner, money transfer business owner, and women selling a variety of items in the mining areas.

After the trained CHWs returned to their work locations, they started informing the people in their work area about the COVID-19 services, though some more proactively than others. The CHWs had different ways to make their new services known. Most CHWs started with informing the persons in their surroundings that, in addition to malaria testing, they now also provided COVID-19 testing. In some cases, they also talked to several key persons in surrounding mining communities. In cases when someone came to test for malaria, it was common to inform the person about the COVID-19 test service and offer him/her a test. In some areas, WhatsApp groups were used for sharing news. These were very effective in spreading the word about the option for COVID-19 testing.

### Reactions to the presence of COVID-19 services in gold mining communities.

#### Positive responses to COVID-19 services.

Reactions to the COVID-19 services were overall positive:

*“The people were very happy because we are at quite a distance from the city. Most people were positive. You will always have some who are not interested, we know that. They walk away or are busy with their phone.”(CHW, area of Grankreek)*

*“They were happy because Maripasoula [location of hospital in FG] is at a distance from here. They were happy that I could give them the information and that there was an option to test.” (CHW, area of Yaw Pasi)*

*“People were enthusiast because at that time we did not yet have self-tests here, and there was a period that many people had flu symptoms. They became afraid and were searching for a place to test, otherwise they would have to go to the city and it is very expensive. They reacted positive because it was free and could be done right there.” (CHW, area of Sarakreek)*

#### Negative responses to COVID-19 services.

Occasionally, negative reactions were encountered, but they were rare:

*“Some people get angry. They say: I am not going to test. I try to calm them down, ease them. And explain calmly why it is good.” (CHW, area of Tjilipasi)*

*“Some were not interested, they said they did not need to know whether they had COVID-19 or not.” (CHW, area of Grankreek)*

Such negative reactions were uncommon though, and generally the CHWs reported that the population in the mining areas was happy and grateful that they were able to perform COVID-19 services in the gold mining areas, nearby and at no cost.

#### Challenges encountered in offering COVID-19 services.

The CHWs encountered several challenges in offering COVID-19 services to the gold mining population, including gold miners’ lax attitudes toward illness in general, their distrust of the CHWs, and the fact that the nasal swab was believed to be painful.

#### Disregard of health advice.

*“Sometimes it is useless to be working with gold miners on issues concerning their health. They always believe they know better than you do, and they call themselves ‘Dr. Pião’ [∼Dr. Digging into the ground]” (CHW, area of Grankreek)*

*“The way the gold miners live together; no mask, no distance, no isolation. Gold miners are not concerned about their health, they see everything as ‘normal.’” (CHW, area of Grankreek)*

*“The gold miners do not listen, they do whatever they want even though you explain everything to them.” (CHW, area of Ampoema)*

#### Self-diagnosis and self-medication.

Related to the relaxed attitude of inhabitants of the mining areas toward health issues is the way that they approach and tackle health issues in general, haphazardly taking medication based on their own diagnosis. A CHW explains:

*“They do not test, they self-medicate. The garimpeiro [Brazilian gold miner] is difficult. When he feels bad, he will first buy medication at the Chinese store, and take that. For example, he takes medication for back ache, and then, when it does not improve, he will take something to cure kidney pains, and so on and so forth, until there is nothing left. Only when nothing helps and he feels really bad, he will go to the doctor” (CHW, Ronaldo)*

Other CHWs confirmed this reading and added that gold miners and others living in the mining areas have little interest in listening to health advice.

### Distrust toward CHWs.

Another challenge, reported by two CHWs, is that some inhabitants of gold mining areas do not trust them, the CHWs, because they are not full medical doctors but rather people from the community.

*“You hear people say: ‘this one does not know anything and then she comes play nurse here’ or ‘from nothing she suddenly became something’. Some people walked away during information sessions.” (CHW, area of Grankreek)*

*“Some, when they are poorly educated, can be aggressive. Some do not believe in our work. They have less trust in us because they already know us.” (CHW, area of Ronaldo)*

### Fear for the nasal swab.

The CHWs also reported that many inhabitants of gold mining areas believed the nasal swabs were painful, affecting their willingness to test. It often took much talking and convincing, reported one of the CHWs:

*“Some were afraid to test, they did not want the stick in their nose. But my wife tried to convince them. She told them that now it is not like in the beginning when everything was new. In the past they really stuck the little stick far inside [your nose], but we do not do that anymore” (CHW, area of Ampoema)*

*“In the beginning people were really afraid because they had tested in the city, where it was very painful, and with some there came blood from their nose. They were placing the stick too deep.” (CHW, area of Sarakreek)*

*“When they came to test for malaria, some also did the COVID test. But others said: ‘…it hurts,’ and I try to explain [to] them that it does not hurt. But they do not come easily [to test].”*

Despite these challenges, the CHWs were highly motivated to serve their communities through the delivery of COVID-19 services.

### Results from the quantitative survey among inhabitants of gold mining areas.

Table 1 presents social and demographic characteristics of inhabitants of gold mining communities who participated in the quantitative survey, both preintervention (March 2022) and postintervention (October 2022). The table shows that with regard to sex, nationality, median age, and profession, the two study groups were similar.

Table 3 presents results for inhabitants of gold mining area in terms of their access to health services, COVID-19 knowledge, perceptions, and practices pre- and postintervention.

**Table 3:** Results from the quantitative survey among inhabitants of gold mining areas, comparing the baseline situation (*N* = 235) with the postintervention situation (*N* = 270)

Indicator	Preintervention (May 2022)	Postintervention (October 2022)
Sample		
* N*	235	270
Female, % (*n*/*N*)	40% (94/235)	34.4% (93/270)
Male, % (*n*/*N*)	60.0% (141/235)	65.6% (177/270)
Access to MoH-MP Services, % (*n*/*N*)		
Who Have Contact Information of the CHW in Their Area	Not Measured	16.3% (44/270)
Who Report That It Is Possible to Conduct a COVID-19 Test in Their Gold Mining Area/Community	Not Measured	28.5% (77/270)
Who Know That There Is a CHW in Their Mining Area Where One Can Conduct a COVID-19 Test	Not Measured	18.1% (49/270)
Knowledge, % (*n*/*N*)		
Able to Name Accurate COVID-19 Transmission Ways	90.2% (212/235)	90.7% (245/270)
Who Know That COVID-19 Cannot Be Transmitted by Bats	44.3% (119/235)	41.1% (119/270)
Able to List One or More COVID-19 Symptoms	96.2% (226/235)	92.2% (249/270)
Four Best Known Measures to Protect Oneself against COVID-19, % (*n*/*N*)		
Naming Face Mask	81.7% (192/235)	63.7% (172/270)
Naming Social Distancing	46.0% (108/235)	53.0% (143/270)
Naming Regularly Disinfecting Hands	69.8% (164/235)	51.1% (138/270)
Naming Regularly Washing Hands with Water and Soap	34.9% (82/235)	35.6% (96/270)
Average Number of Correct Answers Out of Four Statements, % (*n*/*N*)	3 (*N* = 232)	2.9 (*N* = 270)
Who Know That COVID-19 Is NOT Transmitted by Bats	44.3% (104/235)	44.1% (119/270)
Who Are Able to Name At Least One Effective Measure to Protect Oneself against COVID-19 Infection	96.2% (226/235)	93% (251/270)
Attitudes (perceptions), % (*n*/*N*)		
Males Believing Not To Be at Risk of COVID-19 Infection	Not Measured	46.9% (83/177)
Females Believing Not To Be at Risk of COVID-19 Infection	Not Measured	30.1% (28/93)
Reporting To Be Willing To Take a COVID-19 Test at That Moment	86.4% (203/235)	73.0% (197/270)
Practices, % (*n*/*N*)		
Reporting Not Adhering to Any COVID-19 Measures	3.4% (8/235)	56.3% (152/270)
Reporting to Practice Social Distancing	27.7% (65/235)	0% (0/270)
Males Reporting At Least One COVID-19 Infection (regardless of test result)	22.7% (32/141)	25.4% (45/177)
Females Reporting At Least One COVID-19 Infection (regardless of test result)	41.4% (39/94)	44.1% (41/93)
Having Been Tested for COVID-19 At Least Once	48.5% (114/235)	67.8% (183/270)
Surinamese Who Have Been Tested At Least Once	34.8% (16/46)	46.5% (20/43)
Foreign Migrants Who Have Been Tested At Least Once	52.1% (98/188)	71.8% (163/227)
Those Who Had Only Tested Once, Who Reported That the Active-Case Detection Visit of the MoH-MP to the Area Was the Reason They Got Tested	–	15.3% (11/72)
Those Tested Who Named COVID-19 Interventions of the MoH-MP as Their Reason To Get Tested	9.7% (11/113)	12% (22/183)
Those Who Had Tested for COVID-19 At Least Once, Whose Most Recent COVID-19 Test Was Conducted by the MoH-MP Team	25.4% (29/113)	29% (53/183)

CHW = community health worker; MoH-MP = Ministry of Health Malaria Program.

The survey results suggest that differences in COVID-19 knowledge between the two periods were minimal. Generally, the target population was well-informed about COVID-19. The results showed no difference in the extent to which respondents were able to name ways of COVID-19 transmission: 90.2% in the preintervention period versus 90.7% in the postintervention period correctly named one or more ways of transmission. Also, the misperception that bats can transmit COVID-19 did not significantly change after the intervention (44.3% versus 44.1%).

Knowledge of symptoms and ways to protect oneself against COVID-19 infection also remained almost the same. Consistent with the baseline study, the four best-known measures to protect oneself against COVID-19 were wearing a face mask, social distancing, regularly disinfecting hands, and regularly washing hands with water and soap. Even though most respondents were aware that COVID-19 is a dangerous disease that can also affect healthy persons, the data suggest that by the end of 2022, an increased share of the target group doubted the severity of COVID-19. By October 2022, more than half of survey respondents (56.3%; 152/270) reported not adhering to any COVID-19 measures, compared with only 3.4% (8/235) during the survey in May 2022. Also, prior to the intervention, 27.7% (65/235) of surveyed inhabitants of gold mining areas reported practicing social distancing versus not a single person (0%; 0/270) after the intervention. A plausible reason for this trend is that at the moment of the impact study, the pandemic laid dormant: There were very few COVID-19 cases, all restrictions had been lifted, and vaccination programs had come to a virtual standstill.

With regard to risk perception, by October 2022 (postintervention study) 46.9% of men and 30.1% of women believed they were not at risk for COVID-19 infection, even though new COVID-19 cases were still being discovered in Suriname. Inhabitants of the gold mining areas who believed that they were not at risk of contracting COVID-19 supported their response mostly by stating that there was no COVID-19 (anymore) in the mining areas or that the pandemic had passed altogether. Several individuals associated the risk of contracting COVID-19 with their general health and a healthy lifestyle. They would report, for example, that they did not drink alcohol, were in good physical shape, or did not easily get ill. Other reasons for believing not to be at risk of becoming infected with COVID-19 were mentioned less often.

Just over half of respondents believed that there was a risk that they would become infected with COVID-19 (55.9%; 151/270). The main reason for believing to be at risk was simply that there still is COVID-19, followed by the observation that “Anyone can get it.” Compared with men, women were significantly more likely to believe that they were at risk of becoming infected with COVID-19 (men: 50.3%; 89/177 versus women: 66.7%; 62/93; χ^2^, *P* <0.01).

The data suggest that the outreach activities of the CHWs positively affected test behavior. Since the start of the intervention, the number of individuals from gold mining communities who had tested at least once increased from 48.5% (114/235) before the public health intervention to 67.8% (183/270) after the intervention (Table 3). Also, for 12% of respondents, the fact that CHWs had come to their location was a primary reason for getting tested. Among those who had only tested once, 15.3% (11/72) indicated that the reason to get tested was because of the ACD mission of the MoH-MP team to the gold mining area. It is very likely that these people would not have tested without the MoH-MP visit. Furthermore, among persons who had tested for COVID-19 at least once (*N* = 183), 29% had performed their most recent test through one of the services provided by the MoH-MP (53/183).

## STATISTICAL ANALYSES

During the research intervention (May–November 2022), a total of 1,300 persons (927 males and 373 females) were tested for COVID-19, of whom 102 (7.9%) tested positive (Table 2). The majority (88%) of those tested were foreign migrants (Table 4). One hundred and fifty-four persons were tested in the context of contact tracing, of whom 27 (17.5%) were positive. A total of 312 asymptomatic persons were tested, either in the context of contact tracing or during ACD campaigns, of whom seven (2.2%) were positive (Table 2).

**Table 4:** Number of people tested and test results by nationality

Nationality of Persons Tested	Ag-RDT Negative	Ag-RDT Positive	Total Tested (% of total *N*)
Brazilian	1,009	93	1,102 (84.8%)
Surinamese	153	4	157 (12.1%)
Other	36	5	41 (3.2%)
Total	1,198	102	1,300

Ag-RDT = antigen rapid diagnostic test.

Information about vaccination status was available for 939 of the subjects tested for COVID-19. In this group, 66.2% (624/939) received at least one vaccination dose and 46.2% (434/939) were fully vaccinated against COVID-19. Among foreign migrants, 71.4% (612/863) received at least one vaccination dose, and 49.1% were fully vaccinated. Among Surinamese, 13.1% had received at least one vaccination dose (Figure 2).

Apart from COVID-19 testing and dissemination of COVID-19 information, the CHWs continued to perform their malaria test and treat services. During the study period, 3,127 persons were tested for malaria of whom 14 (0.5%) were positive. All were imported malaria cases. Through passive surveillance, 729 were tested by CHWs because of symptoms; 11 of these (1.5%) tested positive. An additional 2,398 persons were tested in ACD campaigns for malaria, of whom three (0.01%) tested positive (Supplemental Table 1).

## DISCUSSION

In general, participants in gold mining areas were satisfied with the provision of COVID-19 services in their communities, especially because it was free and nearby. Lowering test cost, variability in type of testing sites, and provision of culturally tailored testing sites for communities are recommended strategies to address COVID-19 testing hesitancy.^11^

The CHWs encountered several challenges in conducting their COVID-19 outreach work. When explicitly asked if they would be willing to get tested for COVID-19 by a health worker, three-quarters of interviewees responded affirmatively. In practice, however, CHWs experienced that when they offered testing services, inhabitants of the gold mining areas were not very eager to comply, even when displaying symptoms. Probably the most serious impediment was that gold mining populations in Suriname are typically quite nonchalant regarding their health.^6^ They are prone to self-diagnose and self-medicate and will go test or seek medical help only when they feel very ill. This lax attitude toward public health issues resulted in an observed lack of adherence to COVID-19 measures, including quarantining when positive, even at the height of the pandemic.

There were also other reasons that gold mining populations did not want to get tested for COVID-19. In October 2022, the perception that the pandemic was (almost) over and that COVID-19 was now just like the regular flu lessened the willingness to test. The CHWs also reported on people’s fear of the impractical advice of isolation, which would prevent them from working and earning money. Owing to housing and the way of life in gold mining areas, isolation of positive cases was practically impossible. In addition to the advice to isolate, positive cases were strongly encouraged to keep a safe distance and always to use a face mask in the vicinity of others. Another problem was fear of being stigmatized or discriminated against if tested positive. The CHW in Sarakreek, for example, reported an instance where some persons were left out of work because of discrimination after testing positive. Consequences on employment and stigma have previously been listed as barriers to COVID-19 testing.^11^ The overall limited enthusiasm for COVID-19 testing was possibly further reduced by the idea that the nasal swab was painful.

Because of these conditions, CHWs frequently undertook very expensive travel over poor roads for hours to be confronted upon arrival with only one person who was willing to get tested. Nevertheless, with a lot of explaining and patience, the CHWs managed to subdue fears and convince many of those who initially refused to get tested. Thus, despite the many challenges, it is likely that the program reached inhabitants of gold mining areas who would not have tested without the MoH-MP COVID-19 intervention.

We did not observe large outbreaks in the study areas, despite the almost weekly feast gatherings with hundreds of attendees among the gold miners in the study areas and the difficulty of isolating positive cases. An explanation for this could be that outbreaks went unnoticed in this relatively healthy population with vaccination coverage of nearly 50%. It is not likely that gold miners with minor flulike symptoms would seek testing for COVID-19. Health status has been identified as a barrier to testing. It has been described that there are challenges in encouraging individuals who feel healthy to take COVID-19 tests.^11^ On the other hand, testing of asymptomatic individuals also yielded a low percentage of positive cases.

It was often difficult to determine the number of contacts or determine the source of an infection owing to these frequent mass-spreading events attended by positive cases. Moreover, CHWs were not able to do adequate contact tracing for persons tested on the border with French Guiana who were infected in French Guianese gold mines.

The survey results suggest that COVID-19 educational and awareness activities have had minimal effects. Differences between the baseline situation (April 2022) and the postintervention situation (October 2022) with regard to COVID-19 knowledge were negligible. The study results were affected by the pandemic tailing off and illness events becoming, in many cases, relatively benign. By October 2022, COVID-19 was no longer perceived as a severe health problem. In addition, widespread vaccination, especially among the migrant population, fueled the perception that COVID-19 was no longer something to be concerned about. The lack of concern about COVID-19 was reflected in the lack of adherence to protective measures. By the end of the research period, more than half of the respondents reported that they were not doing anything anymore to limit their chances of becoming infected with COVID-19 and no one practiced social distancing.

It has been reported before that CHWs could play an important role in providing health services to populations that otherwise, mostly owing to their remoteness, lacked access to even basic health provisions.^12^^,^^13^ In Suriname, the deployment of CHWs was implemented in 2006, when it was observed that malaria control in mobile and remotely living populations could not be achieved other than with the onsite presence of diagnosis and treatment services. Probably because of the effectiveness of this intervention, the health service provision of these CHWs was readily accepted and resulted in the near elimination of malaria, even among these hard-to-reach populations.^9^ Elsewhere, the services of CHWs have been less appreciated, for several reasons, including that services offered for free were less valued.^14^ However, especially in low- and middle-income countries, it is recognized that health inequities can be reduced and vital bridges between health services and communities can be maintained by formally deploying CHWs.^14^

### Study limitations.

This study had some limitations that, to a limited extent, may have influenced our results. First, poor weather conditions and flooding of some study areas during the initial stages of the study made access temporarily not feasible. This influenced the number of people who could be included during the study period. Also, the remoteness of the working areas impacted verifiability of the implementation of activities. With close supervision and by implementing verification methods such as GPS tracking, we addressed this challenge. Finally, during the intervention time, the number of COVID-19 cases had declined steeply and all national restrictions had been lifted. Because of these changes, the target population was (even) less worried about COVID-19, which likely affected interest in COVID-19 services and willingness to test.

## CONCLUSION

This study shows that integrating COVID-19–related health services in the activities of CHWs working in remote areas is feasible. The expansion of services provided by the CHWs in Suriname gold mining areas was appreciated by the largest share of the target population. The study demonstrated that motivated CHWs, even those with limited formal education, are capable of carrying out a wide array of health-related tasks, including disease diagnostic, treatment, and health communication. In Suriname, the CHWs used their medical training, coupled with knowledge of local health beliefs and behavior, to curb both the malaria and the COVID-19 pandemics. In places such as the remote Suriname gold mining areas, CHWs with their in-depth understanding of the local culture, are indispensable in reaching the highly mobile and hard-to-reach local populations. The findings suggest that by training, capacitating, and supporting CHWs, people in rural, sparsely populated places such as gold mining areas in the global south can access malaria, COVID-19, and other health services that otherwise would remain out of reach.

## Supplemental Materials

10.4269/ajtmh.23-0734 Supplemental Materials
