# Supplementary material for: Acceptability and Feasibility of Provision of COVID-19 Services by Community Health Workers to Remote Gold Mining Communities in Suriname
Source: Am J Trop Med Hyg. 2024 Aug 27;112(4 Suppl):109–18. doi: 10.4269/ajtmh.23-0734 (PMC11965712; doi:10.4269/ajtmh.23-0734)
Supplement: Supplemental Materials [file tpmd230734.SD1.pdf]

Interviewer Initialen:

Vragenlijst

Inclusie: *Stel deze vragen om te bepalen of de persoon mee kan doen aan het onderzoek.*

Heeft u in 2022 minstens 6 maanden in een goudgebied/garimpo in Suriname gewerkt?
1. Ja 0. Nee (Persoon behoort niet tot de doelgroep. Stop het interview)

Bent u 18 jaar of ouder?
1. Ja 0. Nee (Persoon behoort niet tot de doelgroep. Stop het interview)

1. Datum: ___________________________

2. Locatie : 1. Alimoni
   2. Fafão
   3. Parada Obrigatorio
   4. Curutela de Claudia /Sarakreek
   5. Kraboedoin
   6. Vila Brazil
   7. Agua Branca
   88. Anders: _____________________________

A. GEGEVENS OVER DE RESPONDENT

3. Geslacht (omcirkel het juiste antwoord) : 0 = Vrouw 1 = Man

4. Leeftijd:

5. Geboorteland.
   1. Suriname
   2. Braziliё
   3. Guyana
   4. China
   5. Dominicaanse Republiek
   6. Cuba
   88. Anders, namelijk:_____ _________________________

6. Wat is op dit moment uw <u>belangrijkste</u> werk of bezigheid in het goudgebied / deze garimpo? [MAAR 1 ANTWOORD MOGELIJK]
   1. Goudzoeker (*porcentista* / *gowtuman*)
   2. Machinehouder /Baas / dono de máquina
   3. Sekswerker
   4. Transport (ATV/Boot/Auto)
   5. Graafmachine operator
   6. Kok / Kokkin
   7. Eigenaar van winkel of bar
   8. Vrouw van goudzoeker, zelf geen inkomen
   9. Monteur / *mecánico*
   10. Verkoopt spullen (geen vaste plek)/ *Marreteiro/a*
   88. Anders, namelijk: ___________________

7. Wanneer bent u begonnen met werken in de goudsector/garimpo, ongeacht het land?
   1. 2022 (Dit jaar)
   2. 2020-2021 (~1 tot 2 jaar geleden)
   3. Tussen 3 en 5 jaar geleden (2018-2020)
   4. Tussen 6 en 10 jaar geleden (2013-2017)
   5. Meer dan 10 jaar geleden (vóór 2013)

B. GEZONDHEID ALGEMEEN

8. Over welke **TWEE** gezondheidsproblemen **bent u** het meest bezorgd wanneer u in de garimpo bent? LEES DE ANTWOORDEN NIET VOOR. TWEE ANTWOORDEN MOETEN GEKOZEN WORDEN.
   1. Werkgerelateerde verwondingen/ongelukken
   2. Kanker
   3. SOAs inclusief HIV/aids
   4. Malaria
   5. COVID-19
   6. Problemen met de nieren
   7. Problemen met de lever
   8. Rugklachten
   9. Leishmania (busjaws)
   10. Huidproblemen/-infecties / - schimmel
   11. Hoge bloeddruk / Hypertensie
   12. Diabetes
   88. Anders: ____________________
   888. Anders: _____________________

9. Welke **TWEE** gezondheidsproblemen ziet u het meest bij mannen in de garimpo waar u nu werkt?
LEES DE ANTWOORDEN NIET VOOR. TWEE ANTWOORDEN MOETEN GEKOZEN WORDEN
   1. Werkgerelateerde verwondingen/ongelukken
   2. Kanker
   3. SOAs inclusief HIV/aids
   4. Malaria
   5. COVID-19
   6. Problemen met de nieren
   7. Problemen met de lever
   8. Rugklachten
   9. Leishmania (busjaws)
   10. Huidproblemen/-infecties / - schimmel
   11. Hoge bloeddruk / Hypertensie
   12. Diabetes
   88. Anders: ___________________
   888. Anders: ____________________

10. Welke **TWEE** gezondheidsproblemen ziet u het meest bij vrouwen in de garimpo waar u nu werkt?
LEES DE ANTWOORDEN NIET VOOR. TWEE ANTWOORDEN MOETEN GEKOZEN WORDEN
   1. Werkgerelateerde verwondingen/ongelukken
   2. Kanker
   3. SOAs inclusief HIV/aids
   4. Malaria
   5. COVID-19
   6. Problemen met de nieren
   7. Problemen met de lever
   8. Rugklachten
   9. Leishmania (busjaws)
   10. Huidproblemen/-infecties / - schimmel
   11. Hoge bloeddruk / Hypertensie
   12. Diabetes
   88. Anders: ___________________
   888. Anders: ____________________

Kunt u aangeven welke van de onderstaande gezondheidsproblemen u zelf ervaren heeft in 2022?	Ja	Nee	Weet niet/ weigert
11. Werkgerelateerde verwondingen/ongelukken			
12. Hoge bloeddruk / Hypertensie – *pressão alta*			
13. Diabetes – *diabetes*			
14. Hoog cholesterol - *colesterol alto*			
15. Leishmania			
16. Malaria			
17. COVID-19 – *COVID o Corona*			
18. Rugklachten - *dor nas costas*			
19. Pijn in nieren - *dor nos rins*			
20. Pijn aan lever - *dor no fígado*			
21. Huidproblemen/-infecties /-schimmel – *problemas de pele como infecção ou fungo*			

22. Hebt u dit jaar (2022) antibiotica gebruikt?
   0. Nee **>>> Go To 25**
   1. ja
   99. Weet niet/Weigert

23. **Indien Q22=ja**: Hoe hebt u deze antibiotica gekocht of verkregen? GEEF OPTIES:
   1. Op dokters recept
   2. Zelf gekocht in de apotheek / farmácia
   3. Iemand die verkoopt in de garimpo
   4. Van iemand gekregen
   88. Anders: ____________________________

24. Toen u de antibiotica innam, hebt u toen de hele kuur (*cartela*) afgemaakt zonder onderbrekingen?
   0. Nee, ik heb slechts een deel gebruikt, en een deel niet
   1. Nee, ik gebruik af en toe een paar pillen, met tussenpauzes
   2. Ja, kuur in 1x af gemaakt
   88. Anders: _____________

Alleen vrouwen >40: *preventivo / teste de Papanicolau*

25. Wanneer heeft u voor het laatst een uitstrijkje laten maken?
   0. Nog nooit
   1. 2022
   2. 2020-2021
   3. 2018-2019
   4. 2017 of eerder (5 of meer jaar terug)
   99. Weet niet

26. Het malariaprogramma heeft mensen in de goudvelden getraind die de malariatest kunnen doen. Weet je waar je zo iemand kunt vinden in deze garimpo, of hier dichtbij?
    0. Nee, geen idee
    1. Ik denk wel dat er iemand is, maar ik weet niet wie of waar
    2. Ja, ik weet wie, maar ik weet niet hoe ik de persoon kan bereiken.
    3. Ja, ik ken de persoon en weet waar de persoon is of heb een telefoonnummer.
    88. Anders: ____

C. KENNIS OVER COVID-19

27. Weet u hoe iemand besmet kan raken met COVID-19? (*COVID, Corona*) Wat is de oorzaak? [LEES DE ANTWOORDEN NIET VOOR. OMCIRKEL ALLE ANTWOORDEN DIE WORDEN GENOEMD]
    1. In de buurt van iemand zijn die besmet is/ Binnen 1.5 of 2 meter van besmette persoon zijn.
    2. In dezelfde (gesloten) ruimte zijn met een besmette persoon.
    3. Door de lucht; virussen/deeltjes in de lucht.
    4. Als een besmette person niest in je gezicht/ dichtbij je.
    5. Als je een object of oppervlak aanraakt dat aangeraakt is door een besmette persoon.
    88. Anders: ____
    99. Weet niet

28. Hoe weet iemand dat hij of zij COVID-19 heeft? Wat zijn de symptomen van COVID-19? [LEES DE ANTWOORDEN NIET VOOR! OMCIRKEL ALLE ANTWOORDEN DIE GENOEMD WORDEN]
    1. Koorts - *uma febre*
    2. Keelpijn - *dor de garganta*
    3. Hoesten - *tosse*
    4. Ademhalingsproblemen - *problemas respiratórios*
    5. Hoofdpijn - *dor de cabeça*
    6. Lichaamspijn - *dor no corpo*
    7. Verlies van geur en/of smaak - *Perda de olfato e/ou paladar*
    8. Griep / griepachtige verschijnselen
    9. Vermoeidheid – *cansaço*
    88. Anders: ____________________
    99. Weet niet.

Bent u het eens of oneens met deze uitspraken? - *teses*	Eens	Oneens	Weet niet
29. COVID-19 is een heel besmettelijke ziekte			
30. Vleermuizen kunnen je COVID-19 geven			
31. COVID-19 is niet gevaarlijk, het is als een griep (*gripe*)			
32. Alleen mensen die al verzwakt zijn door ziekte of ouderdom overlijden aan COVID-19			
33. Je kunt COVID-19 krijgen door iets aan te raken dat door een besmette person is aangeraakt, en daarna in je ogen te wrijven.			

34. Wat kan iemand doen om zich tegen COVID-19 te beschermen? (algemeen) [LEES DE ANTWOORDEN NIET VOOR! OMCIRKEL ALLE ANTWOORDEN DIE GENOEMD WORDEN]
    1. Je kunt niets doen / je kunt je niet beschermen.
    2. Vaccinatie.
    3. Een gezichtsmasker dragen.
    4. Social distancing - 1.5 tot 2 m.
    5. Handen regelmatig met een ontsmettingsmiddel of alcohol sprayen.
    6. Handen regelmatig met water en zeep wassen.
    7. Huismiddeltjes of bosmedicijn gebruiken, zoals thee van bladeren of met knoflook/gember.
    8. In het bos/de garimpo blijven, niet naar de stad gaan.
    88. Anders; ____

35. Wat doet **u zelf <u>nu nog</u>** om uzelf tegen COVID-19 te beschermen?
[LEES DE ANTWOORDEN NIET VOOR! OMCIRKEL ALLE ANTWOORDEN DIE GENOEMD WORDEN]
1. Niets (meer)
2. Ik ben gevaccineerd
3. Ik ga niet naar drukke plaatsen/feestjes.
4. Ik draag een gezichtsmasker wanneer ik naar de stad ga.
5. Ik blijf uit de buurt van mensen met COVID-19 of griep verschijnselen.
6. Ik spuit/wrijf mijn handen regelmatig met een ontsmettend middel / alcohol.
7. Ik blijf in het bos/ de garimpo; Ik ga zo weinig mogelijk naar de stad.
8. Ik gebruik medicijnen van de apotheek (farmácia)
9. Ik gebruik huismiddeltjes of bosmedicijn.

88. Anders; ____

D. Risiko perceptie

36. Denkt u dat u op dit moment risico loopt om besmet te raken met COVID-19?
0. Nee
1. Ja **>> Go To 38**
2. Alleen een heel klein risico **>> Go To 38**
99. Weet niet

37. **Indien Q36=Nee** : Waarom denkt u dat u geen COVID-19 zult krijgen?
MEERDERE ANTWOORDEN MOGELIJK, LEES NIET VOOR.
1. Er is (bijna) geen COVID-19 meer
2. Er is (bijna) geen COVID in de garimpo
3. Ik leef gezond
4. Ik ga niet naar veel plaatsen.
5. Ik ga (bijna) niet naar de stad
6. Ik ben gevaccineerd
7. Ik neem huismiddeltjes/bosmedicijn
88. Anders: _____________

>> GoTo 39

38. **Indien Q36=Ja** : Waarom denkt u dat (een klein) risico loopt om COVID-19 te krijgen?
MEERDERE ANTWOORDEN MOGELIJK, LEES NIET VOOR
1. Er is nog steeds COVID-19
2. Iedereen kan het krijgen
3. Mensen die besmet zijn gaan niet in isolatie
4. Ik bescherm mezelf niet

88. Anders: ____

D. Ervaring met COVID-19

39. Hebt u ooit COVID-19 gehad?
0 Nee, **>> SKIP NAAR SECTIE E. Q44**
1 Ja
2 Ik weet het niet zeker, ik denk van niet. **>> SKIP NAAR SECTIE E. Q44**
3 Ik weet het niet zeker, ik denk van wel

Vraag 40-43 ALLÉÉN indien 39 = "Ja" of "Ik denk het wel" (Antwoordopties #1 of # 3);

40. Hoe vaak hebt u COVID-19 gehad? [ ]

41. <u>De laatste keer</u> dat u COVID-19 had, was dat bevestigd door een test? Hebt u positief getest?

0. Nee 1. Ja 99. Weet niet

42. <u>De laatste keer</u> dat u COVID-19 had, bent u toen minimaal 5 dagen in quarantaine gegaan? (QUARANTAINE BETEKENT, GEÏSOLEERD VAN ANDEREN, ZONDER FYSIEK CONTACT MET ANDEREN)

1. Nee   1. Ja **>> Go To 44 , sectie E**   99. Weet niet

43. Indien 42=Nee: Waarom niet? MEERDERE ANTWOORDEN MOGELIJK.
    1. Geld verdienen/Als ik niet werk eet ik niet
    2. Ik ben voorzichtig, ik zorg ervoor dat ik geen anderen besmet
    3. Het is niet mogelijk hier in de garimpo
    4. Er zijn geen plekken hier om in quarantaine te gaan.
    88. Anders: ____

E. TEST GEDRAG

44. Is het mogelijk om hier in deze garimpo, of hier vlakbij, een COVID-19 test te doen?

0. Nee **>> GOTO 46**   1. Ja   66. Weet niet **>> GOTO 46**

45. Waar in of vlakbij deze garimpo kun je een COVID-19 test doen? MEERDERE ANTW. MOGELIJK
    1. MSD
    2. Zelftest
    3. Gezondheidswerkers komen soms naar de garimpo om te testen
    4. Anders, namelijk: ______________________________

46. Waar zou u heen gaan als u op dit moment zou willen testen op COVID-19? LEES DE ANTWOORDEN NIET VOOR. MEERDERE ANTWOORDEN MOGELIJK
    0. Ik zou niet gaan testen
    1. Frans Guiana kliniek of ziekenhuis
    2. BOG
    3. Tropclinic (SUCAN), Geyersvlijt of Anamoestr.
    4. MSD /Malaria team
    5. MZ (interior clinic)
    6. Ik zou een zelf–test doen
    7. Medilab/Brahma
    8. Ziekenhuis
    9. Ik zou naar de stad gaan en daar iemand vragen om me ergens te brengen
    88. Anders: ______________

47. Hebt u wel eens een COVID-19 test gedaan/laten doen?
    VERGEET NIET: <u>OOK ZELFTESTEN EN TESTEN IVM VLIEGREIZEN</u>
    0. Nee **>> GoTo sectie F, Q52**   1. Ja   99. Weet niet

Q 48-51 ALLÉÉN VOOR MENSEN DIE WEL EENS EEN COVID-19 TEST GEDAAN HEBBEN (Q47 = JA)

48. **Indien Q47=JA**: Hoe vaak hebt u een COVID-19 test gedaan? Indien nooit getest: 0

49. **Indien 47=JA**, <u>Waarom</u> hebt u deze COVID-19 test(en) gedaan? MEERDERE ANTWOORDEN MOGELIJK
    1. Internationaal reizen
    2. Ik voelde symptomen/ voelde me niet lekker
    3. Gezinslid was positief getest
    4. Gezondheidswerkers waren gekomen en ze hebben (bijna) iedereen getest.
    88. Anders: ____
    66. NVT, Nooit getest.

50. **Indien Q47=JA**, Waar hebt u uw meest recente test gedaan?
   1. Zelf-test
   2. Tropclinic, Suriname
   3. In de garimpo bij een MSD
   4. In de garimpo toen gezondheidswerkers kwamen
   5. In Suriname, maar kent naam locatie niet
   6. In Brazilië
   7. Kliniek of ziekenhuis in Fr. Guyana
   88. Anders: __________

51. In welk jaar deed u uw meest recente COVID-19 test?

☐ 2020 ☐ 2021 ☐ 2022

F. TEST BEREIDHEID (WEER VOOR IEDEREEN)

52. Indien een gezondheidswerker u nu, op dit moment, zou vragen om een COVID-19 test te doen, zou u dat doen?
   0. Nee 1. Ja >> **GA NAAR SECTIE G. Q54** 99. Weet niet

53. **INDIEN 52 = NEE**, Kunt u me vertellen waarom u niet bereid bent om te testen? (LEES DE ANTWOORDEN NIET VOOR. MEERDERE ANTWOORDEN MOGELIJK)
   1. Ik heb geen symptomen, het is niet nodig.
   2. Er is geen COVID-19 meer.
   3. De testen zijn pijnlijk / Het doet zeer in mijn neus/hoofd.
   4. Als ik positief ben gaan andere mensen me slecht behandelen/discriminatie.
   99. Weet niet
   88. Anders: ____

G. VACCINATIE PERCEPTIES, GEDRAG EN BEREIDHEID

54. Bent u gevaccineerd (tenminste 1 dosis)?
   0. Nee **>>> Go To 56** 1. Ja 99. Weigert

55. INDIEN VR 54= JA, Hoeveel vaccinatie doses heeft u gehad (inclusief booster)?
GEEF SCHATTING INDIEN ONZEKER ☐

56. Waar zou u heen gaan indien u op dit moment (nogmaals) gevaccineerd zou willen worden? LEES DE ANTWOORDEN NIET VOOR. MEERDERE ANTWOORDEN MOGELIJK.
   0. Ik wil niet (nogmaals) gevaccineerd worden
   1. BOG
   2. Zorghotel/Noord
   3. Tropclinic (SUCAN)
   4. MZ klinieken(binnenland)
   5. POC (Politie Opleidings Centrum)/Zuid
   6. Ziekenhuis
   7. Huisdokter/openbare (RGD) poli
   8. Ik zou naar de stad gaan en iemand vragen om me ergens te brengen.
   9. Ik zou informeren / bellen / rondvragen om te weten waar ik heen kan.
   88. Weet niet.

EINDE: BEDANK DE RESPONDENT. GEEF DE TELEFOON KAART

Table 1.

Results of malaria testing performed by RDT and microscopy in the gold mining communities during study period

		Malaria Cases	
	**# Tested**	**Imported (%)**	**Locally Acquired (%)**
**Active Case Detection**	2398	3 (100)	0 (0)
**Passive Case Detection**	729	11 (100)	0 (0)
**Total**	**3127**	**14**	**0**
